# Inhibition of Calcium-Activated Chloride Channel ANO1/TMEM16A Suppresses Tumor Growth and Invasion in Human Lung Cancer

**DOI:** 10.1371/journal.pone.0136584

**Published:** 2015-08-25

**Authors:** Linghan Jia, Wen Liu, Lizhao Guan, Min Lu, KeWei Wang

**Affiliations:** 1 Department of Molecular and Cellular Pharmacology, State Key Laboratory of Natural and Biomimetic Drugs, Peking University School of Pharmaceutical Sciences, Beijing 100191, China; 2 Department of Pathology, Peking University Health Science Center, Beijing 100191, China; 3 Department of Pharmacology, Qingdao University School of Pharmacy, Qingdao 266021, China; University of Hull, UNITED KINGDOM

## Abstract

Lung cancer or pulmonary carcinoma is primarily derived from epithelial cells that are thin and line on the alveolar surfaces of the lung for gas exchange. ANO1/TMEM16A, initially identified from airway epithelial cells, is a member of Ca^2+^-activated Cl^-^ channels (CaCCs) that function to regulate epithelial secretion and cell volume for maintenance of ion and tissue homeostasis. ANO1/TMEM16A has recently been shown to be highly expressed in several epithelium originated carcinomas. However, the role of ANO1 in lung cancer remains unknown. In this study, we show that inhibition of calcium-activated chloride channel ANO1/TMEM16A suppresses tumor growth and invasion in human lung cancer. ANO1 is upregulated in different human lung cancer cell lines. Knocking-down ANO1 by small hairpin RNAs inhibited proliferation, migration and invasion of GLC82 and NCI-H520 cancel cells evaluated by CCK-8, would-healing, transwell and 3D soft agar assays. ANO1 protein is overexpressed in 77.3% cases of human lung adenocarcinoma tissues detected by immunohistochemistry. Furthermore, the tumor growth in nude mice implanted with GLC82 cells was significantly suppressed by ANO1 silencing. Taken together, our findings provide evidence that ANO1 overexpression contributes to tumor growth and invasion of lung cancer; and suppressing ANO1 overexpression may have therapeutic potential in lung cancer therapy.

## Introduction

Lung cancer or pulmonary carcinoma that derives from epithelial cells is generally categorized into small cell lung cancer (SCLC) and non-small cell lung cancer (NSCLC). As the most common type of lung cancer, NSCLC accounts for 84% of the estimated cases with two major subtypes: adenocarcinoma and squamous cell carcinoma [1]. At present, the pathogenesis and development of lung cancer has not been clearly defined. Increasing evidence indicates that ion channels play a significant role in cancer cell proliferation and invasion, and in driving cancer progression at all different stages [2, 3].

Ion channels are water-filled pores and transmembrane proteins present in all living cells, participating in diverse physiological activities integral to excitability, contraction, secretion, cell cycle and metastatic cascades [4, 5]. Normal expression of ion channels is crucial for maintaining Ca^2+^and tissue homeostasis during cellular proliferation and differentiation through governing cellular ion fluxes, regulating cell volume, and generating membrane potential [6, 7]. Chloride channels are important for many biological processes including transepithelial transport for ion fluxes, cell volume regulation, differentiation and apoptosis [8, 9]. Stable and constant cell volume and ion homeostasis during cell proliferation and differentiation are strictly required for cell function and survival [10, 11]. Chloride flux through chloride channels in response to cell swelling is one of the critical mechanisms by which cells restore their volume following osmotic perturbations and stress caused by intensive metabolic activities resulted from water, nonelectrolyte and ion exchange at the epithelial surfaces [12–14]. Dysregulation of ion channel expression becomes epigenetically abnormal in metastatic cancer cells [15–17]. For instance, upregulation of chloride intracellular channel 1 (CLlC1) is involved in colon cancer cell migration and invasion through mediating regulatory volume decrease (RVD) mechanism [18]; and overexpression of chloride channel3 (ClC3) contributes to multiple human carcinomas such as glioma, lung, breast, and cervical tumors [19]. It has also been reported that the rise in intracellular calcium occurring during hypotonic challenge is related to RVD and involved with cancer apoptosis, indicating the interplay between calcium homeostasis and volume homeostasis [20, 21].

Ca^2+^-activated Cl^-^ channels (CaCCs) are major regulators of epithelial secretion and cell volume regulation [22, 23]. TMEM16A, also known as DOG1, ORAOV2, or TAOS-2, was identified from airway epithelial cells as a bona fide CaCC that mediates endogenous Ca^2+^-activated chloride current [24–26]. TMEM16A has also been referred to as anoctamin 1 (ANO1) because of its anion selectivity and eight (OCT) transmembrane segments [25]. The *ANO1/TMEM16A* gene is localized on 11q13, one of the most frequently amplified regions in human cancers [27, 28] and associated with a poor prognosis [29]. It has recently been shown that ANO1/TMEM16A is amplified or overexpressed in several human cancers such as gastrointestinal stromal tumors (GIST), prostate cancer, head and neck squamous cell carcinoma (HNSCC), breast cancer and colorectal cancer cells [27, 30–33]. ANO1 overexpression is also correlated with poor prognosis of HNSCC and breast cancer patients [30, 34], and pharmacological inhibition of CaCC ANO1 activity by CaCCinh-A01 and T16Ainh-A01 can inhibit cancer cell proliferation [32, 35, 36]. Although the reason for high expression of ANO1 in tumors is unclear, several studies have shown that ANO1 is involved in oncogenic signaling by activating EGFR and CAMK pathways to promote cell cycle and cancer progression [30, 37]. It has been reported that ANO1-interacting proteins such as signaling/scaffolding actin-binding regulatory proteins ezrin, radixin, moesin, and RhoA can participate in the regulation of ANO1 function [38, 39]. More recently, ANO1 and EGFR are found to form a functional complex that regulates HNSCC cell proliferation [40]. However, whether ANO1/TMEM16A plays a role in tumor genesis of lung cancer remains unknown.

In this study, we found that CaCC ANO1 is highly upregulated in human lung cancer tissues. ANO1 upregulation was confirmed in different human lung cancer cell lines. Knockdown of ANO1 expression by short hairpin RNA inhibited cellular proliferation, migration and invasion in lung cancer cells GLC82 and NCI-H520. Inhibition of ANO1 also suppressed tumor growth in nude mice implanted with stable transfected GLC82 cells. Our findings provide evidence that membrane ANO1 protein may serve as a potential biomarker and target for diagnosis and therapy of lung cancer.

## Methods

### Cell culture

Normal lung cell line 2BS, lung cancer cell lines A549 and H1299 were gifts from Dr. Hongti Jia at the Department of Biochemistry and Molecular Biology, Peking University Health Science Center. A549 and H1299 cells were originally obtained from American Type Culture Collection (ATCC, Rockville, MD). 2BS cells were originally obtained from the National Institute of Biological Products (Beijing, China). Lung cancer cell lines GLC82 and Calu-3 for adenocarcinoma and NCI-H520 for squamous cell carcinoma were obtained from American Type Culture Collection (ATCC). The 2BS cells were cultured in DMEM (Invitrogen, Carlsbad, USA), and the other cell lines were propagated in RPMI 1640 medium (Invitrogen). The medium were supplemented with 10% fetal calf serum and 1% penicillin-streptomycin. All cells were maintained in medium at 37°C in a humidified atmosphere of 5% CO_2_.

### shRNA transfections and generation of stable cell lines

ANO1 shRNAs and scrambled shRNA plasmids were constructed by GeneChem Co., Ltd. (Shanghai, China). The shRNAs were cloned into the BamHI and HindIII sites of the pGCsi-U6/Neo/GFP vecor, and the loop sequence at the TTCAAGAGA was used. The target sequence of three ANO1 shRNAs were as follows: shRNA1, CGTGTACAAAGGCCAAGTA; shRNA2, GCATCTATTTGACTTGTCT; shRNA3, CGAAGAAGATGTACCACAT. The scrambled sequence was CGAGTGGTCTAGTTGAGAA. For transfection, 8 μg DNA was used per 60 mm plate with Lipofectamine 2000 (Invitrogen) according to the manufacturer’s instructions, and transfection medium was replaced with culture medium 4 hours after transfection. All subsequent experiments were performed at 48–72 h after transfection and repeated in triplicate. To generate stable GLC82 cell line expressing ANO1 shRNA, transfected cells were selected under 800 μg/ml G418.

### Immunohistochemical staining

Surgically resected human cancer tissue samples consisting of 44 cases of lung adenocarcinoma and 40 cases of squamous cell lung carcinoma were obtained retrospectively from Peking University Third Hospital. The tissue samples were fully de-identified before access by us and all the patients were informed and consented for the use of their excised tissue for future research. The tissue sections were incubated in a 60°C dry chamber for an hour before de-paraffinized in xylene three times and hydrated through a graded series of ethanol. Treated in 3% hydrogen peroxide for 10 minutes, the tissues were then boiled in 10 mM citrate antigen retrieval solution (pH 6.0) for 20 minutes using a microwave oven. Immunohistochemical (IHC) staining was carried out by incubating the tissues with the primary antibody to ANO1 (1:100; ab53212, Abcam) at 4°C overnight. After incubating the tissues with goat anti-rabbit IgG-HRP for 30 minutes at 37°C, DAB chromogen system was used for visualization. Scores were calculated by multiplying the intensity (integer between 0 and 3).

### Western blot analysis

Cells were washed three times in ice-cold phosphate buffer solution and lysed in RIPA buffer with 1 X Halt phosphatase and protease inhibitor cocktails (Pierce, Rockford, IL). Samples were quantified using a BCA protein assay kit (Thermo Scientific, USA). Equal amounts of protein (50 μg) were separated using PAGE (8%) and transferred to nitrocellulose membranes. After blocked with Tris-buffered saline (TBS) containing 5% milk, the blotting membrane was incubated overnight at 4°C with rabbit anti-ANO1 (1:1000; Abcam) and rabbit anti -β-actin (1:500; Santa Cruz, CA). The membrane was incubated with an anti-rabbit lgG-HRP secondary antibody (1:5000, Santa Cruz) for an hour at room temperature and visualized using the Immobilon Western HRP Substrate.

### CCK8 cell proliferation assay

Cell proliferation was measured with the Cell Counting Kit-8 (Dojindo Laboratories, Japan). 500 cells per well were inoculated in 96-well plates. 10 μl of the Cell Counting Kit solution were added into each well at 24, 48, 72 and 96 hours after seeded. Then the 96-well plates were incubated for 2 hours at 37°C before the optical density (O.D.) was measured at 450 nm by a microplate reader.

### Colony formation assay in culture

For colony formation in culture, transfected cells (treated with 800 μg/ml G418 for 2 days after 48 hours of transfection) were plated at 200 cells per well in 6-well plates and incubated in RPMI-1640 medium containing 10% fetal bovine serum for 12–15 days. The remaining colonies were stained with 0.1% crystal violet and counted, and images were taken after staining.

### Soft agar colony formation assay

Colony formation assay in soft agar was carried out in a 6-well plate. The base layer was made by mixing 1.2% agarose (Invitrogen) and equivalent volume of 2× medium with 20% FBS. Then cells from each group were harvested and suspended in medium containing 0.4% agarose and plated over the base layer in triplicate at a density of 3000 cells per well. After 30 days, the clones were observed apparently and the cells were iced for four hours, and then stained by 0.02% crystal violet. Images were taken after staining.

### Wound-healing assay

To measure the migration activity, cells were inoculated in six-well plates, grown to 90% confluence in RPMI-1640 medium containing 10% fetal bovine serum. Then the cells were starved by changing the medium to serum free RPMI-1640 and cultured for 24 h. Cells were scraped with 100 μl plastic pipette tips and washed with phosphate buffer solution. Images were collected every 24 hours by an inverted phase contrast microscope (Olympus; magnification: 10×). The ratio of the remaining wound area was calculated relative to the initial wound area and normalized to scrambled shRNA group or DMSO group.

### Transwell assay

Cell invasion activity was evaluated in 8.0 μm pore size transwell 24-insert plate chambers (Corning, Acton, MA, USA) coated with BioCoatMatrigel (BD Biosciences, Bedford, MA, USA). After pre-starved for 24 hours by culturing in serum free medium, 3X10^4^ (NCI-H520) or 5X10^4^ (GLC82) cells per well were plated in the upper chambers with serum free medium and incubated for 48 (NCI-H520) or 72 (GLC82) hours. The lower chambers were filled with 10% fetal bovine serum medium. After wiping the cells off from the upper side of the upper chamber, the lower side of the upper chamber was fixed with methanol and stained with 4,6-diamidino-2-phenylindole (DAPI; Sigma). Images were photographed under microscope. The DAPI staining area in the lower chamber was normalized to scrambled shRNA group or DMSO group, indicating its relative invasiveness.

### *In vivo* xenograft tumor growth

Purchased from the Department of Laboratory Animal Science of Peking University Health Science Center, 5-week-old female balb/c nu/nu mice were quarantined for 1 week prior to their use in the study. Animals (8 animals per cage) were housed in microisolat with free access to standard rodent chow and water under temperature-controlled conditions (23 ± 2°C) at 70% humidity [33]. Animals were maintained on a reverse 12 h/12 h light/dark cycle (lights on at 7:00 AM). The animal experimental protocols were approved by the Animal Use and Care Committee of Peking University and were consistent with the Ethical Guidelines. GLC82 stable cells (1 x 10^7^ cells per mouse) were obtained from American Type Culture Collection (ATCC, Rockville, MD). GLC82 cells were transfected by scrambled shRNA, ANO1 shRNA1 and ANO1 shRNA2, respectively, and selected by G418 for two weeks before use. GLC82 cells were inoculated hypodermically into the right forelimbs of the nude mice, and in each group eight animals were used. On the 7th day after injection, the tumor sizes were measured every 2–4 days for a period of 2 weeks and calculated by (L x W^2^)/2 (L and W represented the longest longitudinal and transverse diameter, respectively). The endpoint of the experiments was on the 19th day after injection of GLC82 cells to make sure that tumor mass did not significantly interfere with normal body functions of mice or cause pain. Mice were euthanized by intraperitoneal injection of pentobarbital (120mg/kg) combined with 0.25% lidocaine before tumors were removed. Representative pictures were obtained before tumors were weighed using an electronic balance.

### Statistical analysis

The GraphPad Prism 5 was used to analyze data. All data are expressed as mean ± s.e.m. Student’s *t*-test was used in the data analysis of cellular experiments between two groups. The ANOVA was applied to analyze the protein levels of ANO1 in several cell lines, and to compare the difference in tumor growth. For the analysis of human pathologic tissue collections, the Chi-square test was performed. The value of *P*<0.05 was considered to be statistically significant.

## Results

### Overexpression of ANO1 in human lung adenocarcinoma tissues

**Fig 1:**
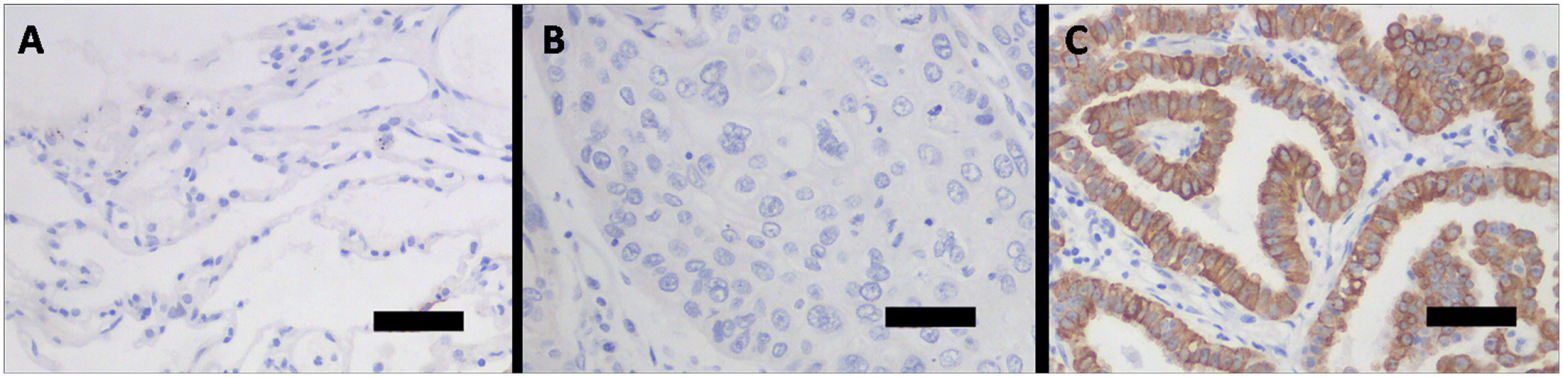
Immunohistochemical staining of ANO1 protein expression in human lung tissues of benign alveoli adjacent to carcinoma, squamous cell carcinoma and adenocarcinoma. (A) Benign alveoli adjacent to carcinoma showing negative ANO1 staining. (B) Squamous cell lung carcinoma showing negative ANO1 staining. (C) Human lung adenocarcinoma cancer tissue showing ANO1 staining (brown color) of neoplastic epithelium. The scale bar indicates 50 μm.

To examine the expression level of calcium-activated chloride channel ANO1 proteins, we used ANO1 antibody for immunohistochemical staining and detected ANO1 expression in lung pathologic tissue specimens from 84 patients. As shown in Fig 1, ANO1 protein was highly expressed in adenocarcinoma of lung, whereas tissues from benign alveoli adjacent to carcinoma and squamous cell carcinoma showed negative staining of ANO1. The analysis of immunohistochemical staining revealed that ANO1 protein expression was positive in 34 of 44 (77.3%) human lung adenocarcinoma tissue samples (Table 1). In tissue specimens from squamous cell lung carcinoma, 6 of 40 (15%) were stained ANO1 positive. These results indicate that ANO1 protein is overexpressed in tumorigenesis of human lung cancer and in particular, the lung adenocarcinoma.

**Table 1:** Correlation between ANO1 expression and lung cancer genesis from human tissue samples.

	Total No.of Samples	No. of ANO1 Positive Samples	% Positive (No./Total)	P value
**Adjacent tissues of cancer**	84	0	0	
**Squamous cell carcinoma**	40	6	15	<0.005^a^
**Adenocarcinoma**	44	34	77.3	

Notes: Samples from 84 lung cancer patients were analyzed. Positive ANO1 staining was seen in 77.3% of adenocarcinoma patient samples examined.

^a^, Chi-square test was used to indicate the statistical significance in the percentage of ANO1 positive staining samples between adjacent tissues of lung cancer or squamous cell carcinoma and adenocarcinoma (p<0.005).

### Upregulation of ANO1 expression in human lung cancer cell lines

**Fig 2:**
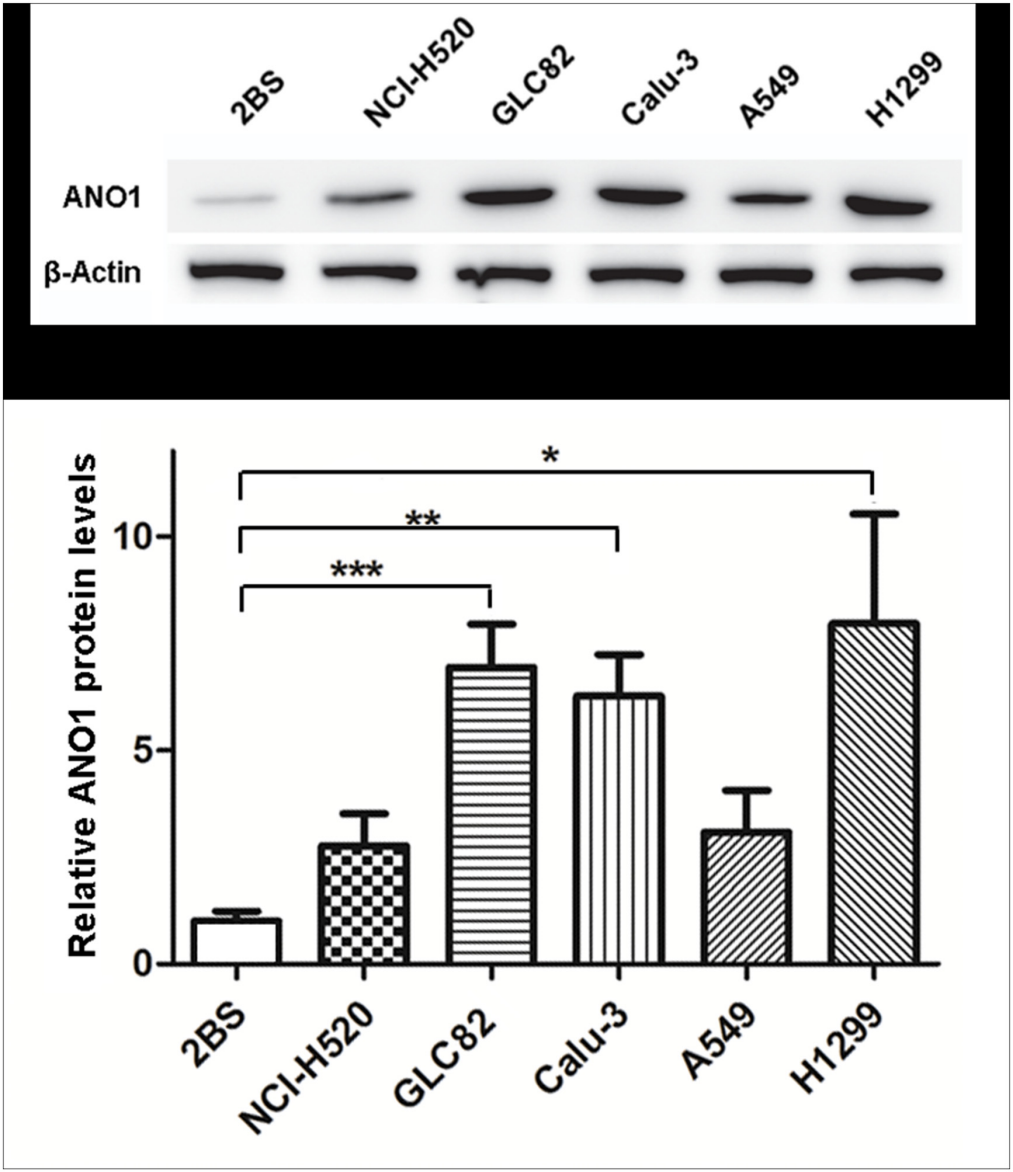
Upregulation of ANO1 expression in human lung cancer cell lines. Top panel: representative western blot images of ANO1 expression in different lung cell lines. Bottom panel: statistical analysis bar chart (n = 3) of ANO1 relative levels in NCI-H520, GLC82, Calu-3, A549 and H1299 cell lines (compared with normal 2BS cells). The expression of ANO1 was normalized to the expression level of β-Actin. ANO1 is significantly overexpressed in GLC82, Calu-3 and H1299 cell lines. Statistical significance by ANOVA is given as *p< 0.05; **p< 0.01 and ***p< 0.001. All data are shown as mean ± s.e.m.

Using immunohistochemistry, our initial finding revealed that ANO1 was overexpressed in human lung cancer tissues especially in adenocarcinoma. To confirm the immunohistochemical finding and determine whether ANO1 is also upregulated in different pathological types of lung cancer cell lines, we selected and tested 6 different cell lines that include the 2BS cells for normal human lung cell line, GLC82 and Calu-3 cells for adenocarcinoma, NCI-H520 cells for squamous cell carcinoma, and A549 and H1299 cells for unconfirmed type of lung cancer (Fig 2). Proteins extracted from 2BS, NCI-H520, GLC82, Calu-3, A549 or H1299 cells were separated by gel electrophoresis under denaturing conditions and probed with ANO1 specific antibody. As shown in the lower panel of Fig 2, quantitative analysis of ANO1 protein expression level showed an elevation about 2.8-fold in NCI-H520, 6.9-fold in GLC82, 6.3-fold in Calu-3, 3.1-fold in A549 and 8.0-fold in H1299, as compared with normal lung 2BS cells. The increase in GLC82, Calu-3 and H1299 cells was statistically significant, but not for NCI-H520 and A549 cells (P = 0.149 and P = 0.238, respectively) although there was a trend of increase in ANO1 expression. This result is consistent with the immunohistochemical analysis of lung cancer tissues obtained from patients (Table 1), further confirming that ANO1 expression is higher in lung adenocarcinoma GLC 82 cells than squamous carcinoma HCI-H520 cells. The GLC82 and NCI-H520 cells that had different expression levels of ANO1 proteins were selected for further investigations.

### Inhibition of GLC82 and NCI-H520 cell proliferation by silencing ANO1

**Fig 3:**
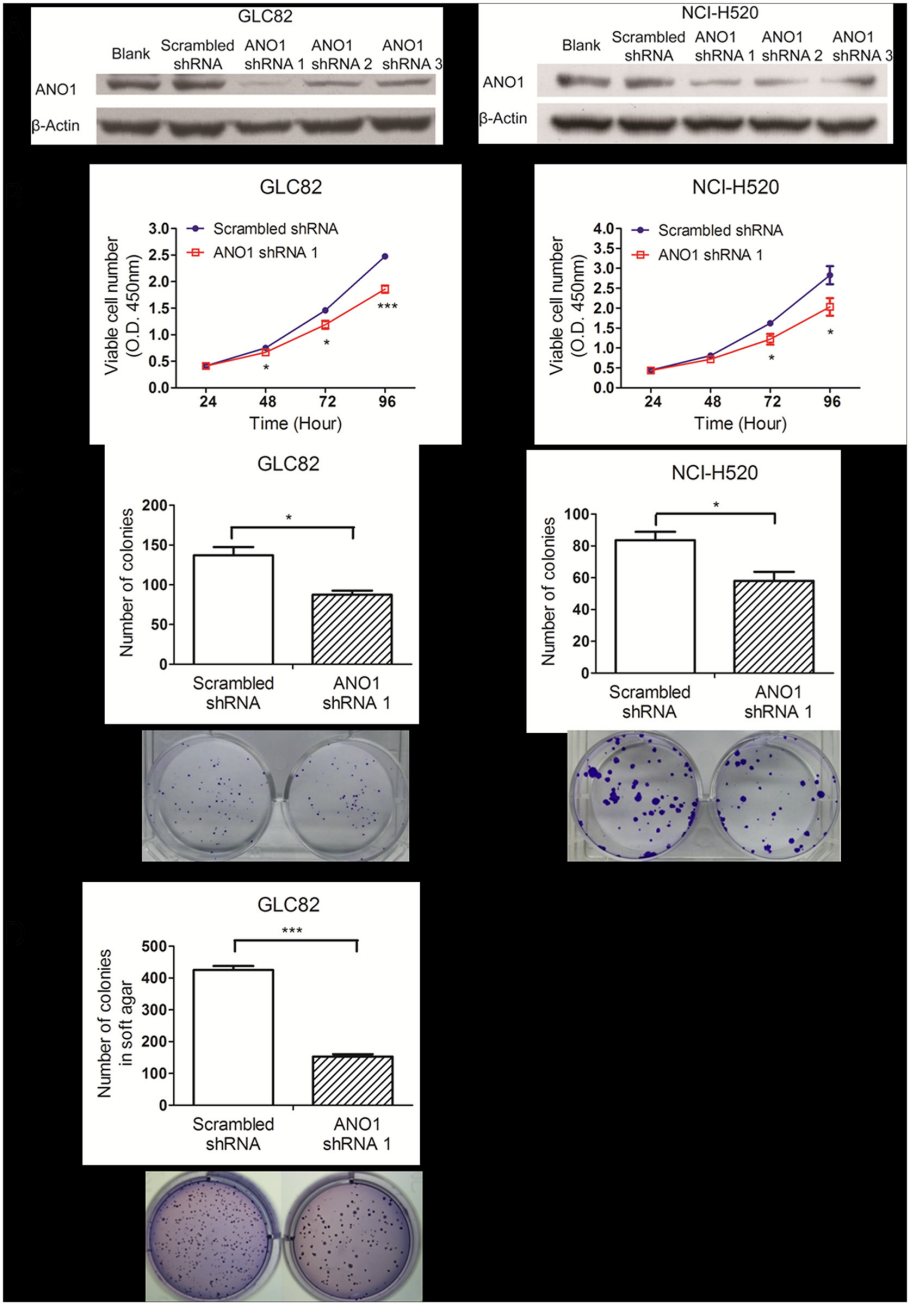
Inhibition of cell proliferation by ANO1 knockdown. (A) RNAi knockdown of ANO1 protein expression in ANO1 expressing cells was shown by Western blot images. The membrane proteins extracted from GLC82 and NCI-H520 cells on the 3rd day after transfection of different ANO1 shRNAs (shRNA1, shRNA2 and shRNA3) were immunoblotted with ANO1 antibody. ANO1 shRNA1 showed the most effective inhibition of ANO1 expression, compared with the other two shRNAs and the scrambled shRNA. (B) GLC82 or NCI-H520 cell proliferation was assessed by CCK8 assay based on the extracellular decreasing of WST8 by NADH produced in mitochondria. Using a microplate reader, the number of cells was quantified by the measurement of absorbance at 450 nm. Transfection of ANO1 shRNA1 resulted in significant inhibition of GLC82 and NCI-H529 cell viability in time-dependent manner as compared with cells treated with scrambled shRNA (n = 6). (C) Cell proliferation of carcinoma cells was assessed by the colony formation assay in culture in the presence of ANO1 shRNA1. Quantitative analysis of ANO1 silencing group showed less colony genesis, compared with scrambled shRNA group (n = 3). Representative images are showed below bar charts. (D) RNAi of ANO1 inhibits the clonogenicity of GLC82 cells in soft agar (n = 3). NCI-H520 failed to form colonies in soft agar. Statistical significance by Student’s *t*-test is indicated as *p< 0.05; **p< 0.01 and ***p< 0.001. Data are expressed as mean ± s.e.m.

To evaluate the biological role of ANO1 in lung cancer cell proliferation, we used shRNAs to knockdown the expression of ANO1 in different cell lines. The effectiveness of three shRNAs was evaluated by Western blot in GLC82 and NCI-H520 lung cancer cells. Compared with transfection of scrambled shRNA, ANO1 shRNA1 transfection was most effective in silencing endogenous expression of ANO1 proteins in both GLC82 and NCI-H520 cells (Fig 3A), and thus it was used for the rest of experiments.

To determine the viability and proliferation, we carried out cell proliferation CCK8 assay using GLC82 and NCI-H520 cells. As shown in Fig 3B, silencing ANO1 significantly slowed down the growth of lung adenocarcinoma GCL82 and lung squamous carcinoma NCI-H520 cells. Proliferation of GLC82 cells was inhibited in time-dependent manner from 89.1% at day 2, 81.6% at day 3 to 75.0% at day 4. Proliferation of NCI-H520 cells was also inhibited from 89.0% at day 2, 75.3% at day 3 to 71.7% at day 4. To further confirm these results, we used two assays of colony formation in both culture dish and soft agar. As shown in Fig 3C, silencing ANO1 resulted in a decrease of 36.2% colony formation in GLC82 cells and 30.7% colony formation in NCI-H520 cells, as compared to scrambled shRNA control. To further confirm the effect of silencing ANO1 on proliferation, we used the soft agar colony formation assay that monitors anchorage-independent cell growth. GLC82 cells could form colonies in soft agar in thirty days, but NCI-H520 cells failed to form colonies. As shown in Fig 3D, proliferation of GLC82 cells treated with ANO1 shRNA was significantly inhibited as compared with the scrambled shRNA group. These results indicate that silencing endogenous ANO1 can inhibit proliferation of GLC82 and NCI-H520 lung cancer cells.

### Reduction of GLC82 and NCI-H520 cell migration and invasion by silencing ANO1

**Fig 4:**
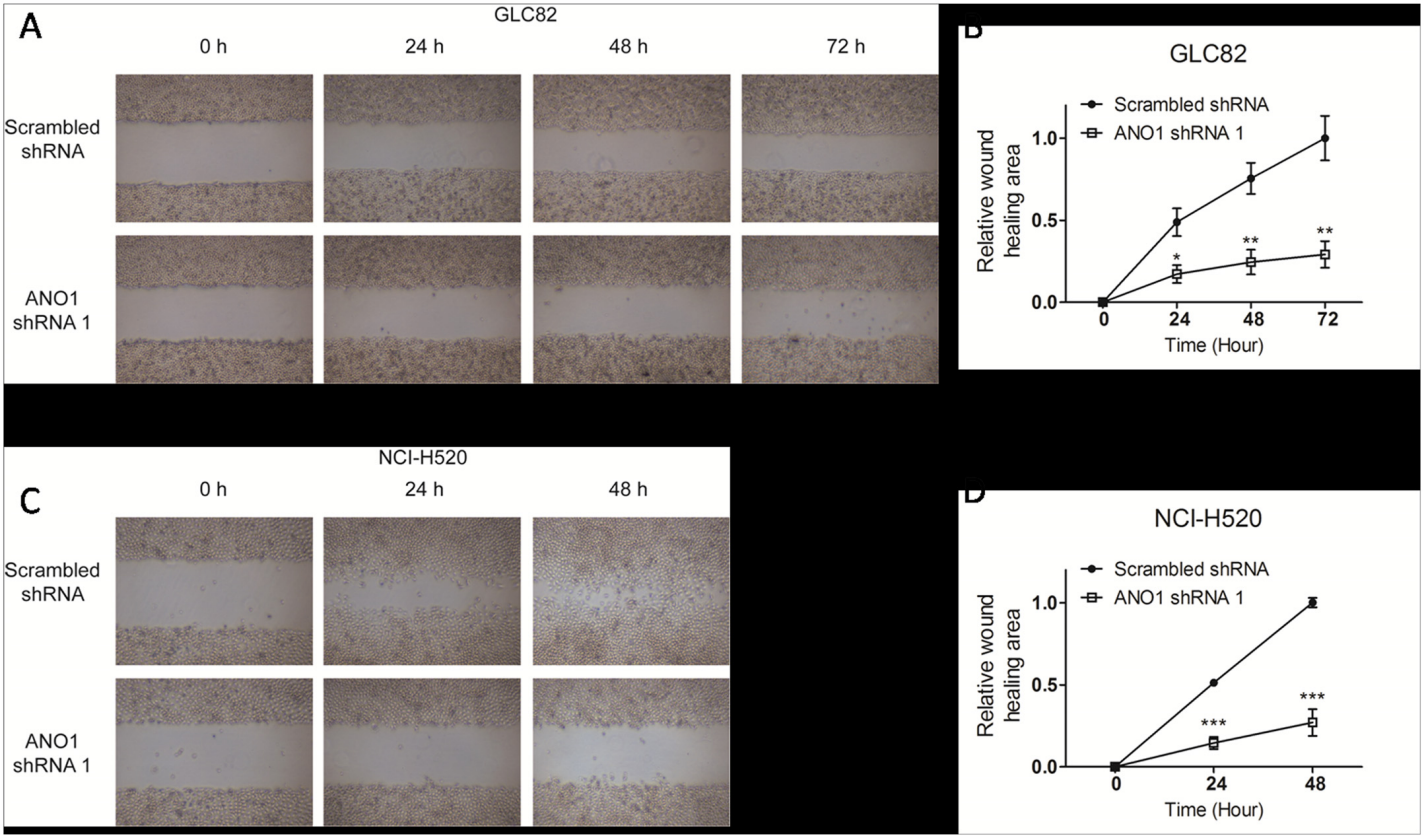
Suppression of lung cancer cell migration by ANO1 silencing in wound-healing assay. Migration of GLC82 and NCI-H520 cells transfected with ANO1 shRNA1 or scrambled shRNA was assessed by wound-healing assay. Two days after transfection, cells were planted in a six-well plate until 90% confluent and then starved for 24 hours. The cell layer was carefully wounded by sterile tips, and incubated with serum free medium. (A) Bright field images of wound at time points 0 h, 24 h, 48 h and 72 h (GLC82) were shown under low magnification. (B) Quantification for the change in wound-healing of GLC82 cells was displayed in line graph and normalized to scrambled shRNA group. (C) Photos of NCI-H520 wound-healing experiment at 0 h, 24 h and 48 h were presented. (D) Line graph of NCI-H520 cells showing cell migration that was inhibited by ANO1 shRNA1 knockdown (n = 3). Statistical significance (Student’s *t*-test) is indicated as *p< 0.05; **p< 0.01 and ***p< 0.001. All data are shown as mean ± s.e.m.

To investigate the migration of lung cancer cells, we utilized wound-healing assay that evaluates wound closure. Two days after transfection, cells were planted in a six-well plate until 90% confluent before starved for 24 hours. The cell layer was carefully wounded by sterile tips, incubated with serum free medium. As shown in Fig 4A and 4B, transfecting GLC82 lung cancer cells with ANO1 shRNA inhibited the wound filling about 66.8% at 24 h, 67.5% at 48 h and 74.3% at 72 h, as compared with scrambled shRNA. In NCI-H520 lung cancer cells (Fig 4C and 4D), the wound-healing in ANO1 knockdown group was only about 29.1% at 24 h and 27.6% at 48 h as compared with the scrambled shRNA control group. At the 48 h, the wound-healing was almost complete in the scrambled shRNA control group. These results show that endogenous ANO1 promotes tumor cell migration, and silencing ANO1 inhibits the migration of lung cancer cells.

**Fig 5:**
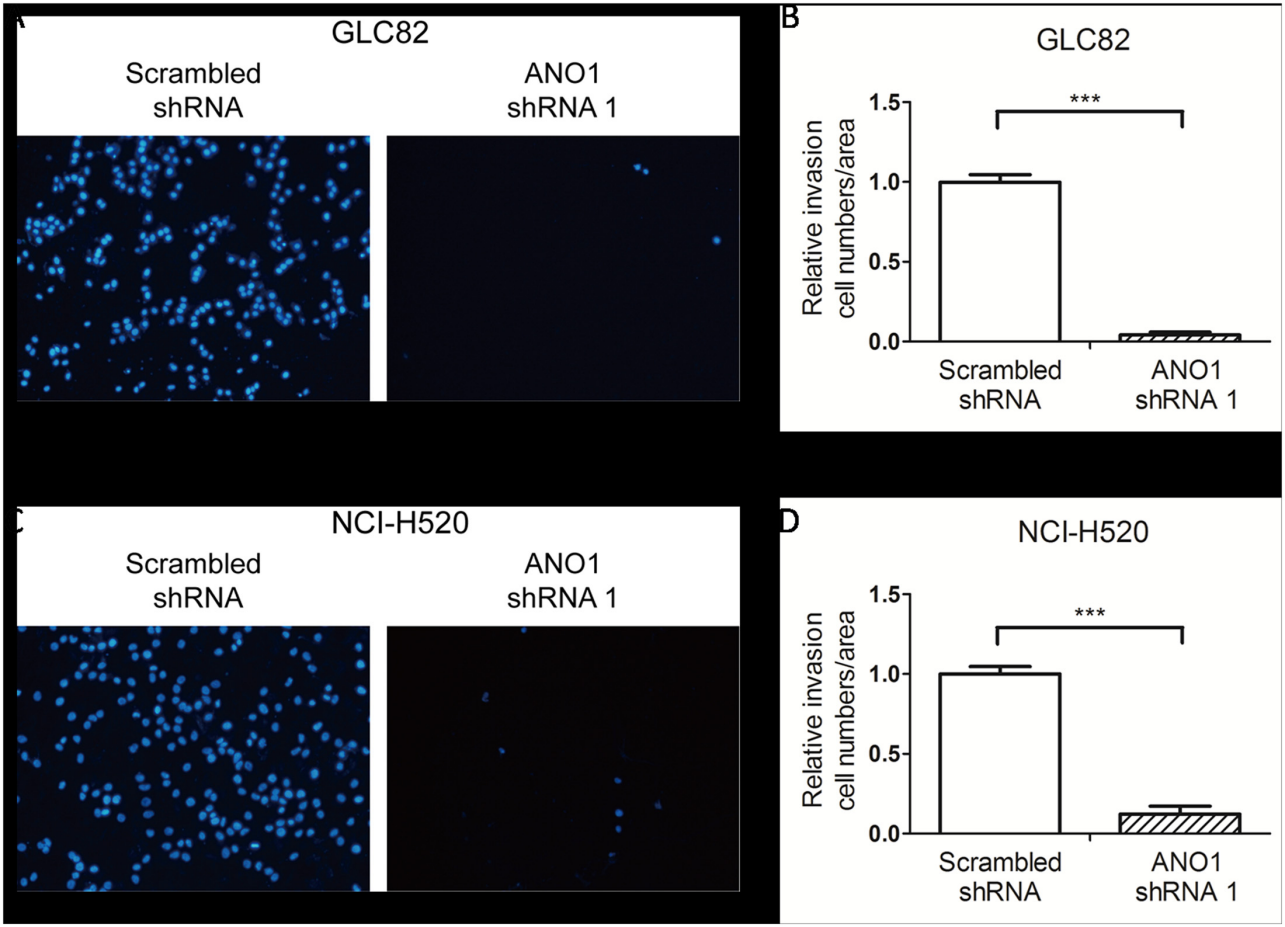
Inhibition of cell invasion by silencing endogenous ANO1 in transwell assay. GLC82 and NCI-H520 cells were starved for 24 hours before incubated in the upper chamber with Matrigel transwell filters. After 48 hours (NCI-H520) or 72 hours (GLC82), cells invading into the lower chamber were fixed with methanol and stained with DAPI. (A) Images represent microscopic fields of the invading GLC82 cells. (B) The invasiveness of cells expressing ANO1 shRNA 1 or scrambled shRNA was quantified by stained area of invading GLC82 cells. The invasion area is normalized to scrambled shRNA group. (C) Representative photos of NCI-H520 cells invaded through Matrigel transwell filters. (D) Bar chat of NCI-H520 cells showing the decrease of cell invasion by ANO1 shRNA1 knockdown (n = 3). Statistical significance by Student’s *t*-test is indicated as *p< 0.05; **p< 0.01 and ***p< 0.001. Data are expressed as mean ± s.e.m.

The most threatening feature of malignancy in lung cancer is the potential for invasion and metastases. To further examine whether silencing ANO1 also affects cell invasion, we used the transwell assay. Two days after transfection with ANO1 shRNA1 or scrambled shRNA, GLC82 and NCI-H520 cells were starved for 24 hours before further incubation in the upper chamber. After 48 hours incubation for NCI-H520 cells or 72 hours incubation for GLC82 cells, cells invading into the lower chamber were fixed with methanol and stained with DAPI. As shown in Fig 5, the invasion potential of tumor cells was dramatically suppressed by ANO1 knockdown. The area of ANO1 silenced GLC82 cells that went through the upper chamber was only 4.0% of the scrambled shRNA group (Fig 5A and 5B). As shown in Fig 5C and 5D, the relative transwell area of ANO1 knockdown in NCI-H520 cells was 12.2%, as compared with cells transfected by scrambled shRNA. These results indicate that silencing ANO1 inhibits the migration and invasion of lung cancer GLC82 and NCI-H520 cells.

### Suppression of xenograft tumor growth in nude mice by ANO1 knockdown

**Fig 6:**
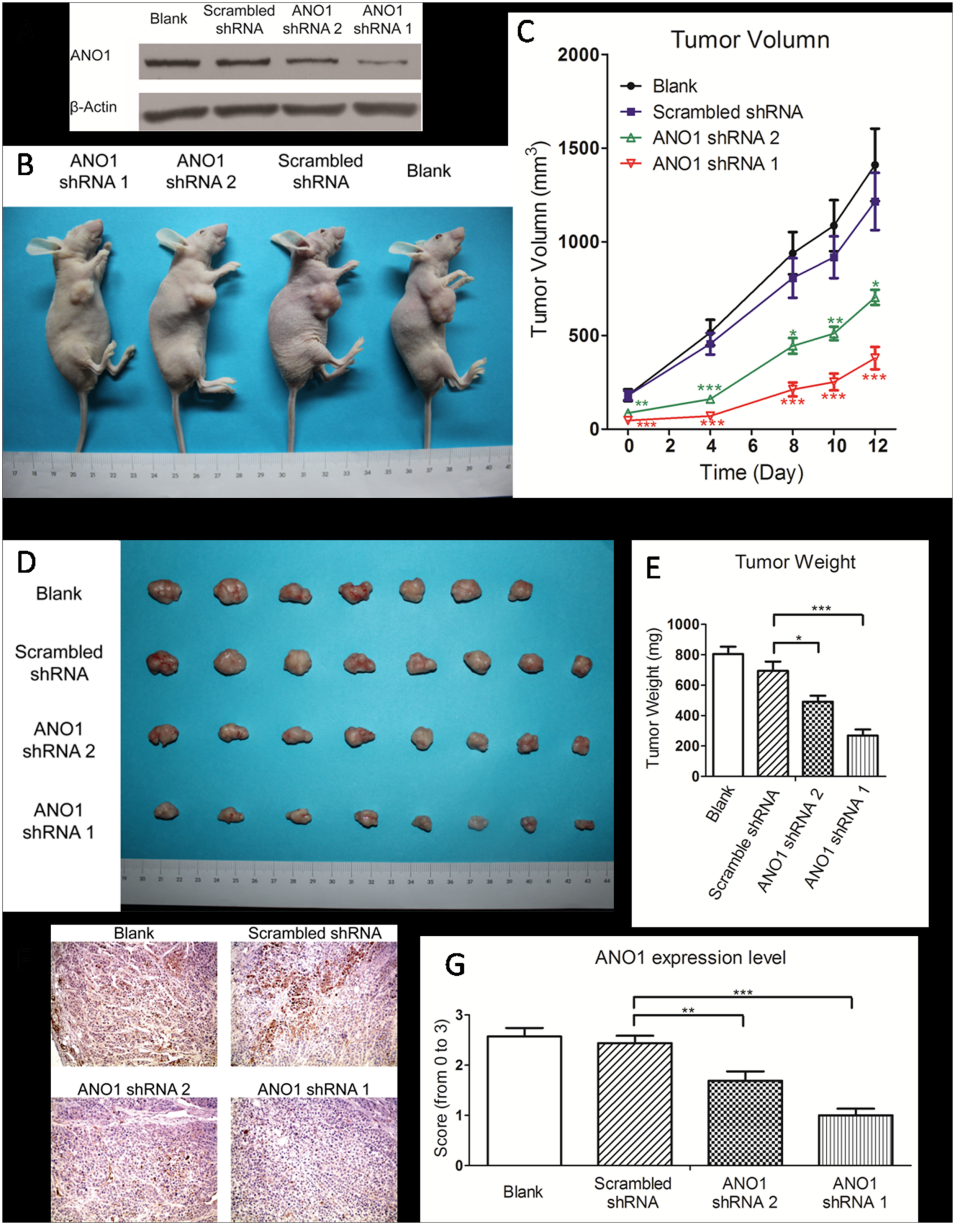
In vivo suppression of xenograft tumor growth by silencing ANO1. Normal GLC82 cells or stable GLC82 cells expressing ANO1 shRNA 1, ANO1 shRNA 2 and scrambled shRNA were inoculated hypodermically into the right forelimbs of 5-week-old female nude mice (1X107 cells per mouse). (A) Western analysis of knockdown efficiency for wild type GLC82 cells and GLC82 cells stable expressing scrambled shRNA, ANO1 shRNA 1 and ANO1 shRNA 2. (B) Representative picture of nude mice carrying GLC82 implanted tumors in four groups: blank group (n = 7), scrambled shRNA group (n = 8), ANO1 shRNA1 group (n = 8) and ANO1 shRNA2 group (n = 8). (C) Tumor size was measured every 2–4 days by Vernier caliper during its development since the 7th day after injection. The growth of tumors expressing ANO1 shRNA1 and ANO1 shRNA2 was significantly inhibited in a time dependent manner, compared with the scrambled shRNA group. (D) Representative picture of tumors picked from mice on the 12th day of generation. (E) Tumors were weighed and analyzed after surgical removal. Comparing with scrambled shRNA tumors, ANO1 shRNAs tumors were lighter. Statistical significance (ANOVA) is shown as *p< 0.05; **p< 0.01 and ***p< 0.001. Data are expressed as mean ± s.e.m. (F) Representative images of immunohistochemical staining of ANO1 expression in xenograft tumor tissues. (G) Analysis of ANO1 expression in xenograft tumor tissues was conducted by scoring from 0 to 3 according to the intensity and area of the staining.

To investigate the effect of ANO1 knockdown on growth of xenograft tumor *in vivo*, three groups of GLC82 cells were individually transfected with ANO1 shRNA1, ANO1 shRNA2 and scrambled shRNAs, and their knockdown efficiency was confirmed by western blot before injection for formation of tumor xenograft in mice (Fig 6A). We injected 10^7^ GLC82 cells per mouse into right forelimbs of 5-week old nude mice in four groups for formation of a tumor xenograft. The size of tumors was first measured 7 days after the injection (as day 0). As shown in Fig 6B and 6C, ANO1 silencing resulted in a significant reduction of tumor growth by 68.8% in shRNA1 group and 42.1% in shRNA2 group, respectively, as compared with the scrambled group at the observation of day 12. At day 12, the tumors were removed from mice and weighted. A remarkable reduction of tumor weight in ANO1 shRNA groups was observed (Fig 6D and 6E). As compared with the control scrambled shRNA, the average tumor weight of ANO1 shRNA1 and shRNA2 groups was about 38.7% and 70.1%, respectively (Fig 6D and 6E). Further immunohistochemical staining of ANO1 in xenograft tumor confirmed that the reduction of ANO1 protein expression levels was consistent with the tumor volume in the groups treated by ANO1 shRNAs with different potency and in the control group (Fig 6F and 6G). These results indicate that ANO1 silencing not only inhibits the migration and invasion of lung cancer cells but also significantly suppresses tumor growth.

## Discussion

The goal of this study was to investigate whether Ca^2+^-activated Cl^-^ channel (CaCC) ANO1 or TMEM16A, originally identified from airway epithelial cells, is involved in progression of non-small-cell lung carcinoma that is typical of epithelial lung cancer.

In this study, we have demonstrated that ANO1 is highly overexpressed in several lung cancer cell lines and human adenocarcinoma tissue samples. Silencing ANO1 suppresses cell proliferation, migration and invasion, and the growth of implanted tumors. These findings highlight the significance of ANO1 membrane protein as a potential biomarker and possible therapeutic target in lung cancer therapy.

The ANO1 or TMEM16A has previously been reported to be overexpressed in many cancers including oral squamous cell carcinoma (OSCC), gastrointestinal stromal tumor (GIST), head and neck squamous cell carcinoma (HNSCC), prostate cancer, breast cancer and pancreatic ductal adenocarcinoma (PDAC) as well as colorectal cancer cells [27, 30, 32, 33, 41–43]. It is noticeable that these carcinomas are mostly epithelium originating tumors, indicating not only a crucial role of ANO1 as an ion channel in sensing and transmitting extracellular signals into intracellular machinery in epithelial cells, but also ANO1 dysfunction in alterations of ion homeostasis and volume regulation in epithelial cancer development and progression.

In this study, our histochemical staining of human samples in this study reveals that 77.3% of lung adenocarcinoma tissues are highly overexpressed with ANO1, whereas only 15% lung squamous cell carcinoma samples show ANO1 positive in the staining. Lung adenocarcinoma and squamous cell carcinoma differ on the basis of histopathological and clinical characteristics and their possible etiologies. Whether such a difference that also relates to their etiology or stage of cancer development is not clear, and requires further examination. Lung squamous cell carcinoma is primarily due to smoking, and adenocarcinoma of the lung is the most common type in non-smokers. Lung adenocarcinoma is usually found in peripheral lung tissues whereas lung squamous cell carcinoma usually originates near a central bronchus [44]. In comparison to squamous cell carcinoma, lung adenocarcinoma shows earlier local invasion and hematogenous metastasis, and has worse prognosis responding to surgical treatment, chemotherapy and radiotherapy [45–47]. The observed difference in ANO1 expression between the adenocarcinoma and squamous cell carcinoma likely highlights the biological differences between these two subtypes, it also suggests different tumor suppressor genes that may be related to the genesis of each histologic type [48, 49].The mechanism underlying ANO1 in proliferation and migration of lung cancer cells is not investigated in this study. It is not quite clear how overexpression or dysregulation of ANO1 contributes to cancer development. To date, evidence has been accumulated indicating that chloride channels are important for control of transepithelial transport for ion homeostasis and cell volume regulation, which is integral to regulation of cell-cycle progression and proliferation [19, 21]. Cell-cycle progression is critically dependent on cell volume in which profound alterations in volume regulation can lead to the generation of apoptosis-resistant cells [50]. The CaCC *ANO1/TMEM16A* gene is located in chromosome band 11q13 that is one of the most frequently amplified regions in human cancer and is associated with a poor prognosis [29, 51]. ANO1 expression is highly correlated with cyclin D1 expression (CCND1) in cells [37, 52], and cyclin D1 is considered to be main driver of the 11q13 amplicon and a key factor of cell cycle for transition from the quiescent G1 phase to the proliferative S phase [53, 54]. This is confirmed by a recent observation that suppression of ANO1 expression leads to reduction of cyclin D1 expression [37, 41]. In addition to the role of ANO1 in cell-cycle progression and proliferation, ANO1 has recently been shown to be involved in oncogenic signaling by activating EGFR and CAMK pathways to promote cancer progression [30], and enhancing MAPK signaling for progression of cell cycle [37]. More recently, ANO1 has been shown to associate with EGFR to facilitate the EGFR-signaling and regulate HNSCC cell proliferation [40]. Therefore, it is likely that ANO1 overexpression in lung cancer results in activation of oncogenic signaling pathways that are partially shared in the pathogenesis of epithelial tumors.

In summary, we have shown in this study that ANO1 is overexpressed in human lung adenocarcinoma tissues and upregulated in lung cancer cell lines. Genetic inhibition of ANO1 expression leads to suppression of proliferation, metastasis and invasiveness *in vitro* and xenograft tumor growth *in vivo*. Therefore, pharmacological inhibition of upregulated ANO1 may provide a strategy and therapeutic potential for treatment of lung cancer or other epithelial cancers.

## Ethics Statement

All authors declare that the use of human cancer tissue samples from patients involved in this research was approved and followed by the guidelines of Ethics Committee of Peking University Health Science Center at Peking University Third Hospital that obtained the informed consent from all patients before surgical procedures. All mice used in this study and animal experimental protocols approved by the Animal Use and Care Committee of Peking University were consistent with the Ethical Guidelines of the Committee.

## References

[1] Detterbeck FC, Boffa DJ, Tanoue LT. The new lung cancer staging system. Chest. 2009;136(1):260–71. Epub 2009/07/09. 10.1378/chest.08-0978 136/1/260 [pii]. 19584208

[2] Pedersen SF, Stock C. Ion channels and transporters in cancer: pathophysiology, regulation, and clinical potential. Cancer Res. 2013;73(6):1658–61. Epub 2013/01/11. 10.1158/0008-5472.CAN-12-4188 [pii]. . 23302229

[3] Tsavaler L, Shapero MH, Morkowski S, Laus R. Trp-p8, a novel prostate-specific gene, is up-regulated in prostate cancer and other malignancies and shares high homology with transient receptor potential calcium channel proteins. Cancer Res. 2001;61(9):3760–9. Epub 2001/04/28. . 11325849

[4] Hille B, editor. Ion Channels of Excitable Membranes: Sinauer, 3rd; 2001.

[5] Aryal P, Sansom MS, Tucker SJ. Hydrophobic Gating in Ion Channels. J Mol Biol. 2014 Epub 2014/08/12. S0022-2836(14)00397-0 [pii] 10.1016/j.jmb.2014.07.030 . 25106689 PMC4817205

[6] Lang F, Foller M, Lang KS, Lang PA, Ritter M, Gulbins E, et al Ion channels in cell proliferation and apoptotic cell death. J Membr Biol. 2005;205(3):147–57. Epub 2005/12/20. 10.1007/s00232-005-0780-5 . 16362503

[7] Razik MA, Cidlowski JA. Molecular interplay between ion channels and the regulation of apoptosis. Biol Res. 2002;35(2):203–7. Epub 2002/11/06. . 12415737 10.4067/s0716-97602002000200011

[8] Zhou ZL, Jiang J, Yin JA, Cai SQ. Identifying interacting proteins of a Caenorhabditis elegans voltage-gated chloride channel CLH-1 using GFP-Trap and mass spectrometry. Sheng Li Xue Bao. 2014;66(3):341–8. Epub 2014/06/27. . 24964852

[9] Duran C, Thompson CH, Xiao Q, Hartzell HC. Chloride channels: often enigmatic, rarely predictable. Annu Rev Physiol. 2010;72:95–121. Epub 2009/10/16. 10.1146/annurev-physiol-021909-135811 19827947 PMC2851227

[10] Okada Y, Maeno E, Shimizu T, Dezaki K, Wang J, Morishima S. Receptor-mediated control of regulatory volume decrease (RVD) and apoptotic volume decrease (AVD). J Physiol. 2001;532(Pt 1):3–16. Epub 2001/04/03. PHY_12151 [pii]. 11283221 10.1111/j.1469-7793.2001.0003g.x PMC2278524

[11] Hoffmann EK, Lambert IH, Pedersen SF. Physiology of cell volume regulation in vertebrates. Physiol Rev. 2009;89(1):193–277. Epub 2009/01/08. 10.1152/physrev.00037.2007 [pii]. . 19126758

[12] Haussinger D. The role of cellular hydration in the regulation of cell function. Biochem J. 1996;313 (Pt 3):697–710. Epub 1996/02/01. 8611144 10.1042/bj3130697 PMC1216967

[13] Lang F, Busch GL, Ritter M, Volkl H, Waldegger S, Gulbins E, et al Functional significance of cell volume regulatory mechanisms. Physiol Rev. 1998;78(1):247–306. Epub 1998/02/11. . 9457175 10.1152/physrev.1998.78.1.247

[14] Furst J, Gschwentner M, Ritter M, Botta G, Jakab M, Mayer M, et al Molecular and functional aspects of anionic channels activated during regulatory volume decrease in mammalian cells. Pflugers Arch. 2002;444(1–2):1–25. Epub 2002/04/27. 10.1007/s00424-002-0805-1 . 11976912

[15] Diss JK, Stewart D, Pani F, Foster CS, Walker MM, Patel A, et al A potential novel marker for human prostate cancer: voltage-gated sodium channel expression in vivo. Prostate Cancer Prostatic Dis. 2005;8(3):266–73. Epub 2005/08/10. 4500796 [pii] 10.1038/sj.pcan.4500796 . 16088330

[16] Fraser SP, Diss JK, Chioni AM, Mycielska ME, Pan H, Yamaci RF, et al Voltage-gated sodium channel expression and potentiation of human breast cancer metastasis. Clin Cancer Res. 2005;11(15):5381–9. Epub 2005/08/03. 11/15/5381 [pii] 10.1158/1078-0432.CCR-05-0327 . 16061851

[17] Parekh AB, Putney JW Jr. Store-operated calcium channels. Physiol Rev. 2005;85(2):757–810. Epub 2005/03/25. 85/2/757 [pii] 10.1152/physrev.00057.2003 . 15788710

[18] Wang P, Zhang C, Yu P, Tang B, Liu T, Cui H, et al Regulation of colon cancer cell migration and invasion by CLIC1-mediated RVD. Mol Cell Biochem. 2012;365(1–2):313–21. Epub 2012/03/20. 10.1007/s11010-012-1271-5 . 22426742

[19] Xu B, Jin X, Min L, Li Q, Deng L, Wu H, et al Chloride channel-3 promotes tumor metastasis by regulating membrane ruffling and is associated with poor survival. Oncotarget. 2014 Epub 2014/12/30. 2966 [pii]. . 25537517 10.18632/oncotarget.2966 PMC4385862

[20] Kerrigan MJ, Hall AC. Control of chondrocyte regulatory volume decrease (RVD) by [Ca2+]i and cell shape. Osteoarthritis Cartilage. 2008;16(3):312–22. Epub 2007/09/15. S1063-4584(07)00256-7 [pii] 10.1016/j.joca.2007.07.006 . 17855127

[21] Kim TH, Kim JS, Kim ZH, Huang RB, Chae YL, Wang RS. Khz (Fusion Product of Ganoderma lucidum and Polyporus umbellatus Mycelia) Induces Apoptosis in Human Colon Carcinoma HCT116 Cells, Accompanied by an Increase in Reactive Oxygen Species, Activation of Caspase 3, and Increased Intracellular Ca. J Med Food. 2014 Epub 2014/12/10. 10.1089/jmf.2013.3135 . 25489715

[22] Huang F, Wong X, Jan LY. International Union of Basic and Clinical Pharmacology. LXXXV: calcium-activated chloride channels. Pharmacol Rev. 2012;64(1):1–15. Epub 2011/11/18. 10.1124/pr.111.005009 [pii]. 22090471 PMC3250081

[23] Hartzell C, Putzier I, Arreola J. Calcium-activated chloride channels. Annu Rev Physiol. 2005;67:719–58. Epub 2005/02/16. 10.1146/annurev.physiol.67.032003.154341 . 15709976

[24] Caputo A, Caci E, Ferrera L, Pedemonte N, Barsanti C, Sondo E, et al TMEM16A, a membrane protein associated with calcium-dependent chloride channel activity. Science. 2008;322(5901):590–4. Epub 2008/09/06. 10.1126/science.1163518 [pii]. . 18772398

[25] Yang YD, Cho H, Koo JY, Tak MH, Cho Y, Shim WS, et al TMEM16A confers receptor-activated calcium-dependent chloride conductance. Nature. 2008;455(7217):1210–5. Epub 2008/08/30. 10.1038/nature07313 [pii]. . 18724360

[26] Schroeder BC, Cheng T, Jan YN, Jan LY. Expression cloning of TMEM16A as a calcium-activated chloride channel subunit. cell. 2008;134(6):1019–29. Epub 2008/09/23. 10.1016/j.cell.2008.09.003 [pii]. 18805094 PMC2651354

[27] Huang X, Gollin SM, Raja S, Godfrey TE. High-resolution mapping of the 11q13 amplicon and identification of a gene, TAOS1, that is amplified and overexpressed in oral cancer cells. Proc Natl Acad Sci U S A. 2002;99(17):11369–74. Epub 2002/08/13. 10.1073/pnas.172285799 [pii]. 12172009 PMC123263

[28] Katoh M. FLJ10261 gene, located within the CCND1-EMS1 locus on human chromosome 11q13, encodes the eight-transmembrane protein homologous to C12orf3, C11orf25 and FLJ34272 gene products. Int J Oncol. 2003;22(6):1375–81. Epub 2003/05/10. . 12739008

[29] Schwab M. Amplification of oncogenes in human cancer cells. Bioessays. 1998;20(6):473–9. Epub 1998/08/12. 10.1002/(SICI)1521-1878(199806)20:6<473::AID-BIES5>3.0.CO;2-N [pii]. . 9699459

[30] Britschgi A, Bill A, Brinkhaus H, Rothwell C, Clay I, Duss S, et al Calcium-activated chloride channel ANO1 promotes breast cancer progression by activating EGFR and CAMK signaling. Proceedings of the National Academy of Sciences. 2013;110(11):E1026–E34. 10.1073/pnas.1217072110 PMC3600458 23431153

[31] Ubby I, Bussani E, Colonna A, Stacul G, Locatelli M, Scudieri P, et al TMEM16A alternative splicing coordination in breast cancer. Mol Cancer. 2013;12:75 Epub 2013/07/20. 10.1186/1476-4598-12-75 [pii]. 23866066 PMC3728142

[32] Sauter DR, Novak I, Pedersen SF, Larsen EH, Hoffmann EK. ANO1 (TMEM16A) in pancreatic ductal adenocarcinoma (PDAC). Pflugers Arch. 2014 Epub 2014/08/29. 10.1007/s00424-014-1598-8 . 25163766 PMC4464647

[33] Liu W, Lu M, Liu B, Huang Y, Wang K. Inhibition of Ca(2+)-activated Cl(-) channel ANO1/TMEM16A expression suppresses tumor growth and invasiveness in human prostate carcinoma. Cancer Lett. 2012;326(1):41–51. Epub 2012/07/24. 10.1016/j.canlet.2012.07.015 S0304-3835(12)00413-2 [pii]. . 22820160

[34] Ruiz C, Martins JR, Rudin F, Schneider S, Dietsche T, Fischer CA, et al Enhanced expression of ANO1 in head and neck squamous cell carcinoma causes cell migration and correlates with poor prognosis. PLoS One. 2012;7(8):e43265 Epub 2012/08/23. 10.1371/journal.pone.0043265 PONE-D-12-13954 [pii]. 22912841 PMC3422276

[35] Berglund E, Akcakaya P, Berglund D, Karlsson F, Vukojevic V, Lee L, et al Functional role of the Ca-activated Cl channel DOG1/TMEM16A in gastrointestinal stromal tumor cells. Exp Cell Res. 2014 Epub 2014/05/16. S0014-4827(14)00194-3 [pii] 10.1016/j.yexcr.2014.05.003 . 24825187

[36] Mazzone A, Eisenman ST, Strege PR, Yao Z, Ordog T, Gibbons SJ, et al Inhibition of cell proliferation by a selective inhibitor of the Ca(2+)-activated Cl(-) channel, Ano1. Biochem Biophys Res Commun. 2012;427(2):248–53. Epub 2012/09/22. doi: 10.1016/j.bbrc.2012.09.022 S0006-291X(12)01762-7 [pii]. 22995309 10.1016/j.bbrc.2012.09.022 PMC3479349

[37] Duvvuri U, Shiwarski DJ, Xiao D, Bertrand C, Huang X, Edinger RS, et al TMEM16A induces MAPK and contributes directly to tumorigenesis and cancer progression. Cancer Res. 2012;72(13):3270–81. Epub 2012/05/09. 10.1158/0008-5472.CAN-12-0475-T [pii]. 22564524 PMC3694774

[38] Wanitchakool P, Wolf L, Koehl GE, Sirianant L, Schreiber R, Kulkarni S, et al Role of anoctamins in cancer and apoptosis. Philos Trans R Soc Lond B Biol Sci. 2014;369(1638):20130096 Epub 2014/02/05. 10.1098/rstb.2013.0096 [pii]. 24493744 PMC3917350

[39] Perez-Cornejo P, Gokhale A, Duran C, Cui Y, Xiao Q, Hartzell HC, et al Anoctamin 1 (Tmem16A) Ca2+-activated chloride channel stoichiometrically interacts with an ezrin-radixin-moesin network. Proc Natl Acad Sci U S A. 2012;109(26):10376–81. Epub 2012/06/12. 10.1073/pnas.1200174109 [pii]. 22685202 PMC3387097

[40] Bill A, Gutierrez A, Kulkarni S, Kemp C, Bonenfant D, Voshol H, et al ANO1 interacts with EGFR and correlates with sensitivity to EGFR-targeting therapy in head and neck cancer. Oncotarget. 2015;6(11):9173–88. Epub 2015/04/01. 3277 [pii]. 25823819 10.18632/oncotarget.3277 PMC4496210

[41] Sui Y, Sun M, Wu F, Yang L, Di W, Zhang G, et al Inhibition of TMEM16A Expression Suppresses Growth and Invasion in Human Colorectal Cancer Cells. PloS one. 2014;9(12):e115443 Epub 2014/12/30. 10.1371/journal.pone.0115443 [pii]. 25541940 PMC4277312

[42] West RB, Corless CL, Chen X, Rubin BP, Subramanian S, Montgomery K, et al The novel marker, DOG1, is expressed ubiquitously in gastrointestinal stromal tumors irrespective of KIT or PDGFRA mutation status. American Journal of Pathology. 2004;165(1):107–13. ISI:000222216000010. 15215166 10.1016/S0002-9440(10)63279-8 PMC1618538

[43] Carles A, Millon R, Cromer A, Ganguli G, Lemaire F, Young J, et al Head and neck squamous cell carcinoma transcriptome analysis by comprehensive validated differential display. Oncogene. 2006;25(12):1821–31. Epub 2005/11/02. 1209203 [pii] 10.1038/sj.onc.1209203 . 16261155

[44] Siegel R, Naishadham D, Jemal A. Cancer statistics, 2013. CA Cancer J Clin. 2013;63(1):11–30. Epub 2013/01/22. 10.3322/caac.21166 . 23335087

[45] Wright JC. Update in cancer chemotherapy, Part III: Lung cancer, Part 1. J Natl Med Assoc. 1985;77(10):815–27. Epub 1985/10/01. 2414458 PMC2571176

[46] Santos-Martinez MJ, Curull V, Blanco ML, Macia F, Mojal S, Vila J, et al [Lung cancer at a university hospital: epidemiological and histological characteristics of a recent and a historical series]. Arch Bronconeumol. 2005;41(6):307–12. Epub 2005/07/02. 13075998 [pii]. 15989887 10.1016/s1579-2129(06)60230-9

[47] Rowell NP, Williams CJ. Radical radiotherapy for stage I/II non-small cell lung cancer in patients not sufficiently fit for or declining surgery (medically inoperable): a systematic review. Thorax. 2001;56(8):628–38. Epub 2001/07/20. 11462066 10.1136/thorax.56.8.628 PMC1746110

[48] Huang F, Zhang H, Wu M, Yang H, Kudo M, Peters CJ, et al Calcium-activated chloride channel TMEM16A modulates mucin secretion and airway smooth muscle contraction. Proc Natl Acad Sci U S A. 2012;109(40):16354–9. Epub 2012/09/19. 10.1073/pnas.1214596109 [pii]. 22988107 PMC3479591

[49] Fan WD, Zhang XQ, Guo HL, Zeng WW, Zhang N, Wan QQ, et al Bioinformatics analysis reveals connection of squamous cell carcinoma and adenocarcinoma of the lung. Asian Pacific journal of cancer prevention: APJCP. 2012;13(4):1477–82. . 22799351 10.7314/apjcp.2012.13.4.1477

[50] Forrest AS, Joyce TC, Huebner ML, Ayon RJ, Wiwchar M, Joyce J, et al Increased TMEM16A-encoded calcium-activated chloride channel activity is associated with pulmonary hypertension. Am J Physiol Cell Physiol. 2012;303(12):C1229–43. Epub 2012/10/05. 10.1152/ajpcell.00044.2012 [pii]. 23034390 PMC3532492

[51] Akervall JA, Jin Y, Wennerberg JP, Zatterstrom UK, Kjellen E, Mertens F, et al Chromosomal abnormalities involving 11q13 are associated with poor prognosis in patients with squamous cell carcinoma of the head and neck. Cancer. 1995;76(5):853–9. Epub 1995/09/01. . 8625189 10.1002/1097-0142(19950901)76:5<853::aid-cncr2820760520>3.0.co;2-6

[52] Huang X, Godfrey TE, Gooding WE, McCarty KS Jr, Gollin SM. Comprehensive genome and transcriptome analysis of the 11q13 amplicon in human oral cancer and synteny to the 7F5 amplicon in murine oral carcinoma. Genes Chromosomes Cancer. 2006;45(11):1058–69. Epub 2006/08/15. 10.1002/gcc.20371 . 16906560

[53] Malumbres M, Barbacid M. To cycle or not to cycle: a critical decision in cancer. Nat Rev Cancer. 2001;1(3):222–31. Epub 2002/03/21. 10.1038/35106065 . 11902577

[54] Malumbres M, Barbacid M. Is Cyclin D1-CDK4 kinase a bona fide cancer target? Cancer Cell. 2006;9(1):2–4. Epub 2006/01/18. S1535-6108(05)00402-2 [pii] 10.1016/j.ccr.2005.12.026 . 16413464

